# Isometric Stretch Alters Vascular Reactivity of Mouse Aortic Segments

**DOI:** 10.3389/fphys.2017.00157

**Published:** 2017-03-16

**Authors:** Sofie De Moudt, Arthur Leloup, Cor Van Hove, Guido De Meyer, Paul Fransen

**Affiliations:** ^1^Laboratory of Physiopharmacology, Department of Pharmaceutical Sciences, University of AntwerpAntwerp, Belgium; ^2^Laboratory of Pharmacology, Faculty of Medicine and Health Sciences, University of AntwerpAntwerp, Belgium

**Keywords:** aorta, contraction, isometric, stretch, preload, relaxation, nitric oxide

## Abstract

Most vaso-reactive studies in mouse aortic segments are performed in isometric conditions and at an optimal preload, which is the preload corresponding to a maximal contraction by non-receptor or receptor-mediated stimulation. In general, this optimal preload ranges from about 1.2 to 8.0 mN/mm, which according to Laplace's law roughly correlates with transmural pressures of 10–65 mmHg. For physiologic transmural pressures around 100 mmHg, preloads of 15.0 mN/mm should be implemented. The present study aimed to compare vascular reactivity of 2 mm mouse (C57Bl6) aortic segments preloaded at optimal (8.0 mN/mm) vs. (patho) physiological (10.0–32.5 mN/mm) preload. Voltage-dependent contractions of aortic segments, induced by increasing extracellular K^+^, and contractions by α_1_-adrenergic stimulation with phenylephrine (PE) were studied at these preloads in the absence and presence of L-NAME to inhibit basal release of NO from endothelial cells (EC). In the absence of basal NO release and with higher than optimal preload, contractions evoked by depolarization or PE were attenuated, whereas in the presence of basal release of NO PE-, but not depolarization-induced contractions were preload-independent. Phasic contractions by PE, as measured in the absence of external Ca^2+^, were decreased at higher than optimal preload suggestive for a lower contractile SR Ca^2+^ content at physiological preload. Further, in the presence of external Ca^2+^, contractions by Ca^2+^ influx via voltage-dependent Ca^2+^ channels were preload-independent, whereas non-selective cation channel-mediated contractions were increased. The latter contractions were very sensitive to the basal release of NO, which itself seemed to be preload-independent. Relaxation by endogenous NO (acetylcholine) of aortic segments pre-contracted with PE was preload-independent, whereas relaxation by exogenous NO (diethylamine NONOate) displayed higher sensitivity at high preload. Results indicated that stretching aortic segments to higher than optimal preload depolarizes the SMC and causes Ca^2+^ unloading of the contractile SR, making them extremely sensitive to small changes in the basal release of NO from EC as can occur in hypertension or arterial stiffening.

## Introduction

During development of hypertension, conduit arteries as well as resistance arteries and arterioles sense elevated wall stresses. In the short and long term, this results in structural and morphological remodeling of the arterial wall. Aside from these “passive” adaptations to increased pressure (Arribas et al., 2006), also the vascular smooth muscle cells (VSMC) of elastic arteries in cross-talk with the endothelial cells (EC) contribute to biomechanical adaptation to hypertension (Fridez et al., 2001, 2002). In hypertensive animals, VSMC are depolarized and the density of voltage-gated Ca^2+^ channels (VGCC) is increased (Pesic et al., 2004; Shi et al., 2015). The voltage-dependence of VGCC window contractions is altered (Fransen et al., 2016) and the preparations spontaneously develop tension (Sekiguchi et al., 1996). Furthermore, the release of endothelium-derived NO is diminished (Sekiguchi et al., 2001) and a pressure- and stretch-activated constrictor response that relies on increased calcium influx through VGCC is elicited (Rapacon-Baker et al., 2001). Chronic effects of hypertension (days, weeks, months) on blood vessels can, however, not be compared with the acute effects of increased stretch, which are far more studied in small than in large arteries (Davis, 2012). Also in the short term however, VSMC contribute to adaptive mechanisms elicited by acute hypertension before the slower geometrical and structural remodeling develop sufficiently to restore the biomechanical environment and function of the arterial wall (Fridez et al., 2001, 2002; Murtada et al., 2016). Acute stretch of large arteries causes changes in contractility in basal conditions or to elevated K^+^ or norepinephrine (Peiper et al., 1974; Wolf and Lentini, 1984; Fischell et al., 1989), and alterations in basal or agonist-stimulated release of NO and cyclooxygenase-derived metabolites from the stretched endothelium (Dainty et al., 1990; Sekiguchi et al., 1996, 2001; Franchi-Micheli et al., 2000). Acute stretch of mouse thoracic aorta elicits two types of intracellular calcium responses in the VSMC. One was a phasic calcium discharge generated by the sarcoplasmic reticulum (SR), the second was a tonic response produced by the activation of stretch-sensitive non-selective cationic channels (NSCC) allowing external Ca^2+^ entry (Fanchaouy et al., 2007). The effects of stretch or preload on vaso-reactivity of mouse aorta is largely unknown and is the topic of the present study.

Most vaso-reactive studies in aortic segments are performed in isometric conditions (constant diameter) and at an optimal preload, which is the preload corresponding to a maximal contraction by non-receptor mediated (high K^+^) or receptor-mediated (α_1_-adrenoceptor) stimulation. In mouse aortic segments, implemented preloads are highly variable among different studies and range from about 1.2–8.0 mN/mm (Fransen et al., 2008, 2015; Van Hove et al., 2009; Ponnoth et al., 2012; van Langen et al., 2012; Lloyd et al., 2013; Taguchi et al., 2014; Gross et al., 2015; Leloup et al., 2015a; Zhou et al., 2015). According to Laplace's law, and assuming a thin, isotropic and homologous wall for large vessels such as the aorta, the transmural pressure (P, in mm Hg) is directly proportional to the preload (PL, in mN) and inversely proportional to its length (l, in mm) and radius (r, in mm): P = PL/(l × 2r) × 7.5) (Syyong et al., 2009; Van Herck et al., 2009; Butlin et al., 2015). Preloads between 1.2 and 8.0 mN/mm roughly correlate with transmural pressures from 10 to 65 mmHg and are sub-physiological. For physiologic transmural pressures around 100 mmHg, preloads of 15 mN/mm should be implemented.

The present study aimed to compare vascular reactivity of 2 mm aortic segments preloaded at “optimal” (16 mN or 8 mN/mm) vs. “(patho) physiological” (20–65 mN or 5–32.5 mN/mm) preloads. Segments were depolarized at different preloads by high extracellular K^+^ to study contractions due to Ca^2+^ influx via VGCC, which can be inhibited with VGCC blockers such as diltiazem (Michiels et al., 2014). They were also stimulated at different preloads with the α_1_-adrenoceptor agonist phenylephrine (PE) to focus on contractions due to intracellular Ca^2+^ release from the IP_3_-sensitive sarcoplasmic reticulum Ca^2+^ store and on contractions due to mixed Ca^2+^ influx from the extracellular space via VGCC and NSCC (Fransen et al., 2015). This study hypothesized that an acute increase of preload would cause VSMC depolarization of the segments, Ca^2+^ unloading of their sarcoplasmic reticulum (SR) and Ca^2+^ influx via stretch-activated cation channels. Further, in light of the higher susceptibility of elastic than muscular arteries to arterial stiffening (Laurent et al., 1994; Ruitenbeek et al., 2008; Borlotti et al., 2012; Zhang et al., 2013) and our recent observations that in mice elastic arteries differ from the smaller muscular arteries by releasing more basal nitric oxide (NO) in baseline conditions (Leloup et al., 2015b), we tested whether endothelium-derived NO –in cross-talk with the subjacent VSMC—modulates arterial compliance at different preloads. Voltage-dependent contractions of aortic segments induced by elevating extracellular K^+^ and contractions by α_1_ adrenergic stimulation with phenylephrine (PE) were studied at different preloads, in the absence and presence of L-NAME to inhibit basal release of NO. Results revealed that contractions at (patho) physiological pressures differ from contractions at optimal pressures with an important role for basal release of NO.

## Methods

### Animals

All animals (C57Bl/6J background) were housed in the animal facility of the University of Antwerp in standard cages with 12–12 h light-dark cycles and free access to regular chow and tap water. Male C57Bl6 mice (*n* = 33) were used, at the age of 5–6 months, and with an average weight of 29.0 ± 0.7 g. Animals were euthanized by perforating the diaphragm while under anesthesia [sodium pentobarbital (Sanofi, Belgium), 75 mg kg^−1^, i.p.]. The thoracic aorta was carefully removed and stripped of adherent tissue. Starting at the diaphragm, the aorta was cut in 6 segments of 2-mm width. Because all segments were cut at 2 mm, preload will be expressed further in mN knowing that to obtain the preload in mN/mm, given preloads have to be devided by 2. Segments were mounted between two parallel wire hooks in a 10 mL organ bath, immersed in Krebs Ringer solution (37°C, 95% O_2_/5% CO_2_, pH 7.4) containing (in mM): NaCl 118, KCl 4.7, CaCl_2_ 2.5, KH_2_PO_4_ 1.2, MgSO_4_ 1.2, NaHCO_3_ 25, CaEDTA 0.025 and glucose 11.1. The solution was continuously aerated with a 95% O_2_/5% CO_2_ gas mixture, to maintain the pH of 7.4, and was regularly replaced to prevent glucose depletion. Isometric contractions and relaxations were measured by means of a Statham UC2 force transducer (Gould, USA). To avoid any vasomotor interference due to prostanoids, 10 μM indomethacin (Federa, Belgium) was present in all experiments. This study was carried out in accordance with the recommendations of the Ethical Committee of the University of Antwerp (2015–52 and 2016–28), and all experiments were performed conform to the Guide for the Care and Use of Laboratory Animals published by the US National Institutes of Health (NIH Publication No. 85–23, revised 1996).

### Isometric preload

According to Laplace's law (Mulvany and Halpern, 1977), P = $\frac{2\pi \;\times \;\text{T}}{\text{IC}\;}$ with P: transmural pressure (mN/mm^2^); *T*: wall tension (mN/mm) or ($T=\frac{PL}{2l}$) with PL, preload (mN) and l, the length of the aortic segment; IC: internal circumference (mm), the transmural pressure can be approximated as $P=\frac{\pi \;\times \;PL}{IC\;\times \;l}$ with PL the preload in mN applied to the segment. In the present study, 2-mm aortic segments were mounted at different preloads of 10, 16, 20, 28, 30, 40, 50, and 65 mN, corresponding to an estimated transmural pressure of respectively 5.8, 8.5, 10.0, 12.8, 13.4, 16.0, 18.2, and 20.7 mN/mm^2^, or 43, 64, 75, 96, 100, 120, 136, and 156 mmHg.

**Figure 1:**
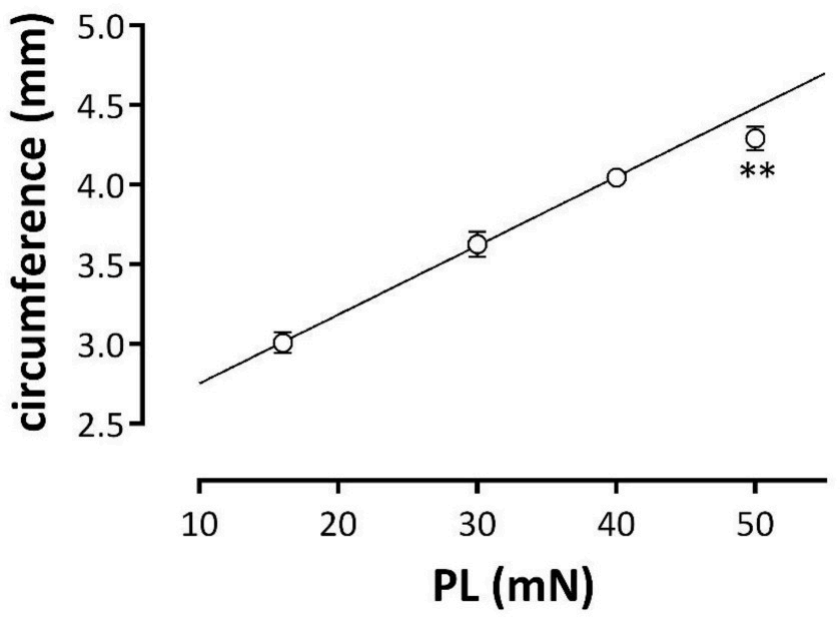
**Relationship between the estimated aortic external circumference and preload (PL)**. Aortic segments were clamped at preloads of 16, 30, 40, and 50 mN and aortic circumferences were determined. Data at 16, 30, and 40 mN were fitted with linear regression (line: circumference (mm) = 0.04^*^PL + 2.32). The estimated diameter at 50 mN was lower than calculated (^**^p < 0.01, *n* = 11).

The internal circumference (IC) of the aortic segments was determined as *IC* = (2 × d) + (π × k) with d, the distance between upper and lower hook as measured with a Zeiss stereoscope (Zemi 2000) with ocular divisions and k, the diameter of one hook (300 μm). IC depends on the preload at which the segments are incubated in the organ bath (Figure 1). From 16 to 40 mN there is a linear relationship between preload and calculated aortic IC with slope of 43 ± 2 μm/mN. At 50 mN, the IC of 4.29 ± 0.07 mm is smaller than extrapolated from this linear relationship (4.49 ± 0.05 mm, *p* < 0.01, *n* = 11). The inner circumference at 30 mN, which translates to a normal transmural pressure of about 100 mm Hg, is 3.63 ± 0.08 mm, which agrees very well with the inner circumference of 3.77 mm measured by ultrasound (Kassab, 2006).

### Experimental protocols

Before subjecting the segments to different experimental conditions to obtain the vasoreactive measurements described below, the aortic segments were stretched and stabilized to one of the following preloads of 10, 16, 20, 28, 30, 40, 50, or 65 mN in Krebs Ringer solution at 37°C. Aortic segments of the same mouse were stretched to different preloads, and measurements were performed in parallel. Randomization of the segments was employed to exclude non-specific effects of the changes in aortic physiology between the proximal and distal segments of the thoracic aorta.

K^+^ concentration-contraction curves (KCC) were measured at different preloads by gradually increasing external K^+^ from 5.9 to 10, 15, 20, 25, 30, 35, 40, and 50 mM by isosmotic replacement of Na^+^ by K^+^ in Krebs Ringer solution. Concentration-contraction curves for α_1_ adrenoceptor stimulation of the aortic segments with phenylephrine (PECC) were cumulatively determined for concentrations of 3 × 10^−9^ to 3 × 10^−6^ M. Both KCC and PECC were fitted with sigmoidal concentration-response equations with variable slope (GraphPad Prism), which revealed maximal responses (Emax) and (the logarithm of) the concentration resulting in 50% of the maximal contraction (logEC_50_ for PE and EC_50_ for K^+^).

Phasic contractions by PE were measured by adding 1 μM PE to the segments incubated for 3 min in Krebs Ringer solution without extracellular Ca^2+^ and with 1 mM EGTA. These contractions are mediated by the IP_3_-dependent release of contractile Ca^2+^ from the SR (Fransen et al., 2015). After the phasic contraction, extracellular Ca^2+^ (3.5 mM) was added to measure the tonic contraction induced by Ca^2+^ influx in the PE-sensitized segment. Next, 35 μM diltiazem was added, to block the Ca^2+^ influx via voltage-gated Ca^2+^ channels. In this way, the contribution of VGCC and NSCC to the tonic contraction can be ascertained (Fransen et al., 2015). In some experiments, segments were incubated for 5 min with levcromakalim (1 μM), a K_ATP_ channel agonist, to repolarize aortic segments to the K^+^ equilibrium potential.

To assess the basal release of NO, contractions by elevated K^+^ or PE were measured in separate segments of the same mouse in the absence and presence of 300 μM L-NAME to inhibit eNOS. We have previously shown that this is the most sensitive way to measure basal NO release (van Langen et al., 2012; Leloup et al., 2015b). Relaxation of 1 μM PE-precontracted segments by endogenous and exogenous NO release was measured by constructing concentration-relaxation curves for acetylcholine (ACh, 3 × 10^−9^ to 3 × 10^−6^ M) and diethylamine NONOate (DEANO, 3 × 10^−10^ to 3 × 10^−6^ M), fitting the curves with sigmoidal concentration-response equations with variable slope (GraphPad Prism) to obtain E_max_- and logEC_50_-values.

### Data analysis

Data are expressed as mean ± sem with n, the number of mice. Aortic segments are always alternated between the different set-ups such that every experimental condition occurred at least in segment position along the thoracic aorta. Data obtained at different preloads or in the presence or absence of L-NAME were compared by one-way ANOVA or two-way ANOVA with Bonferroni multiple comparison post-test (GraphPad Prism). A 5% level of significance was selected.

## Results

### Optimal preload for vascular reactivity

In an initial series of experiments (*n* = 5), the optimal preload was determined by measuring maximal contractile force at preloads of 8, 12, 16, 20, and 24 mN. Basal release of NO was inhibited with 300 μM L-NAME, followed by both receptor-independent (50 mM K^+^) and receptor-dependent (1 μM PE, i.e., an α_1_-adrenoceptor agonist) stimulation of mouse aortic segments. Maximal contractions were measured in the segments preloaded at 16–20 mN. At lower and higher preloads the contraction was smaller. Therefore, a preload of 16–20 mN was chosen as the “optimal” preload to study isometric contractions (data not shown, but see also **Figure 7B**).

### Depolarization with elevated K^+^

**Figure 2:**
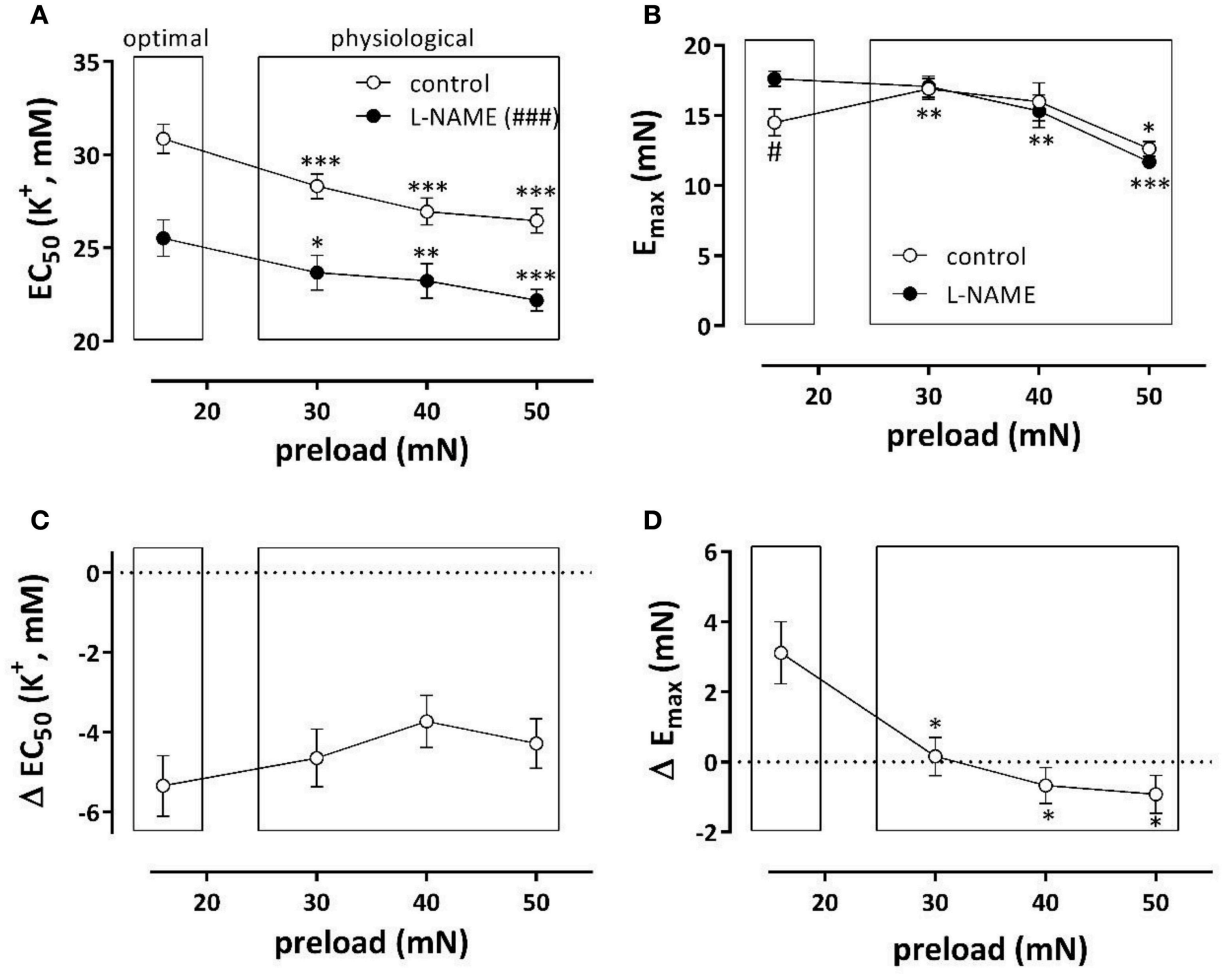
**Depolarization of aortic segments at different preloads**. EC_50_ **(A)** and E_max_ **(B)** values of K^+^ concentration-response curves for depolarization of aortic segments at different preloads in the absence (eNOS active, white) and presence of 300 μM L-NAME (eNOS inactive, black). The shifts of EC_50_ and E_max_ caused by inhibition of eNOS with 300 μM L-NAME are shown in **(C,D)**. ^*^p < 0.05, ^**^p < 0.01, ^***^p < 0.001: 30, 40, or 50 vs. 16 mN; #*p* < 0.05, *###p* < 0.001 control vs. L-NAME, *n* = 7.

Depolarization of aortic segments with elevated K^+^ causes receptor-independent depolarization of the VSMC-membrane, and a resultant isometric contraction mediated by VGCC Ca^2+^ influx [5]. K^+^ concentration-contraction (KCC) curves were constructed at optimal preload of 16 mN, and compared with KCC at 30, 40, and 50 mN (corresponding to physiological pressures of 100–140 mm Hg). Figure 2A shows the half maximal effective K^+^ concentration (EC_50_) for K^+^ at these preloads in the absence and presence of L-NAME, an inhibitor of eNOS. In both conditions, EC_50_ at the physiological preloads shifted to lower K^+^ concentrations, indicating that compared to the optimal preload the sensitivity of the VGCC-mediated contraction increased with higher preload. As expected, in the absence of basal release of NO (presence of L-NAME), the maximal contraction (E_max_) (Figure 2B) at optimal preload significantly decreased at physiological preloads. In the presence of basal NO release, however, a biphasic response was observed, with a new optimal preload at 30 mN, and then a decrease in E_max_ up to 50 mN.

To reveal the role of basal NO release on contractions induced by depolarization with K^+^, Figures 2C,D display the shifts of EC_50_ and E_max_ as a result of eNOS inhibition. The shifts of EC_50_ with L-NAME were not load-dependent (Figure 2C). The increase of E_max_ by eNOS inhibition was only evident at the preload of 16 mN. Results of these experiments indicate that basal NO release has only minor effects on the preload-dependent isometric contractions induced by depolarization with K^+^ elevation at physiological preloads.

### Depolarization of aortic segments at physiological preload

**Figure 3:**
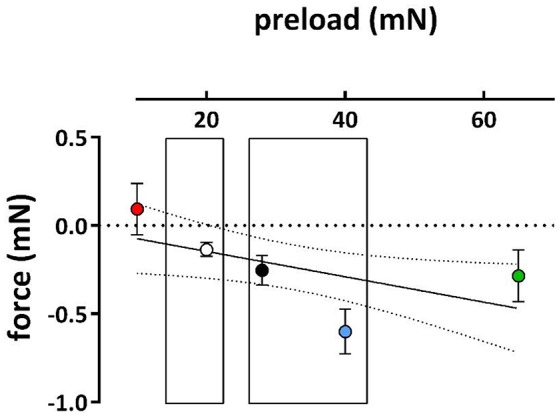
**Effects of removal of external Ca^2+^ on basal force at different preloads**. Decrease of basal force upon removal of extracellular Ca^2+^ in segments initially preloaded at 10, 20, 28, 40, and 65 mN (different symbol colors). (*n* = 5).

Because hypertension has been described to cause depolarization of the resting membrane potential of VSMC (Pesic et al., 2004; Shi et al., 2015), we tested whether a physiological or super-physiological preload of the aortic segments of normotensive mice also caused depolarization. Depolarization of segments in baseline conditions is accompanied by higher influx of Ca^2+^ via the window of VGCC and higher basal tension (Fransen et al., 2012a). Therefore, if segments at high preload are depolarized, it is expected that removal of external Ca^2+^ in these conditions elicits a larger decrease in basal force (Figure 3). Accordingly, there was no decrease of basal force at 10 mN, whereas removal of Ca^2+^ significantly decreased basal force at the higher preloads, suggesting elevated baseline Ca^2+^ influx at the higher (patho) physiological preloads.

**Figure 4:**
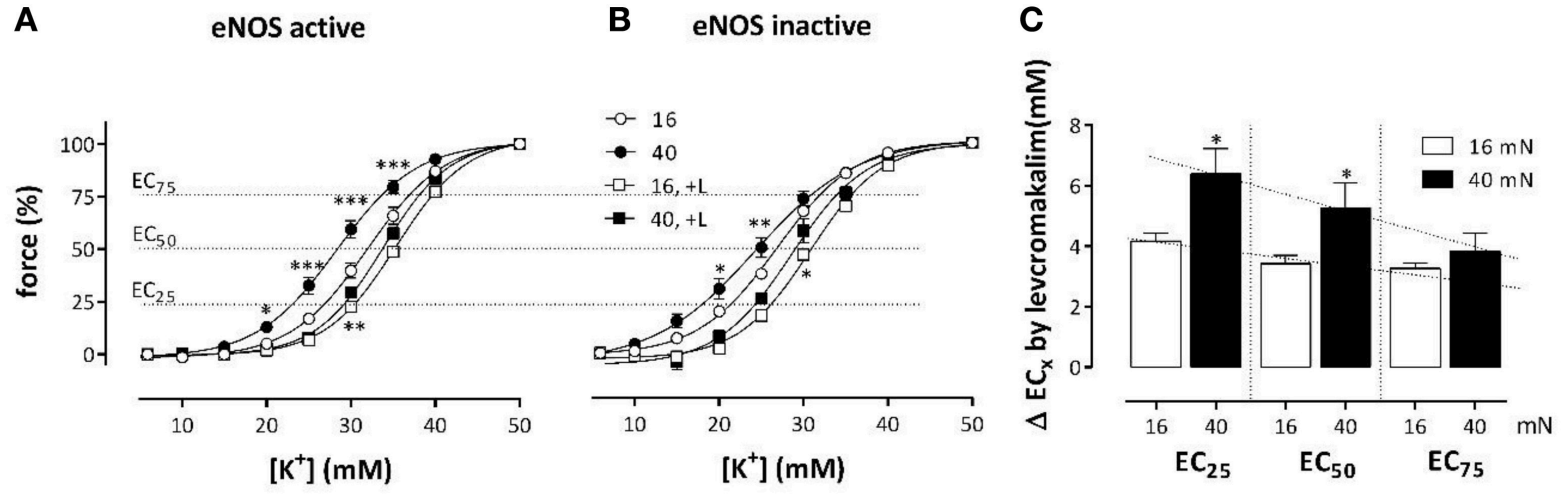
**Effects of levcromakalim on K^**+**^ concentration-contraction curves at 16 and 40 mN**. K^+^ concentration-contraction curves are shifted more to the right by levcromakalim at the preload of 40 mN (black) than at 16 mN (white). Isometric contractions were measured at elevated external K^+^ in the absence (circles) and presence (squares) of 1 μM levcromakalim and in the absence **(A)** and presence **(B)** of 300 μM L-NAME. In **(C)** the absolute shifts of the EC of K^+^ by levcromakalim are shown at 25, 50, and 75% contraction (EC_25_, EC_50_ and EC_75_). Because shifts in the absence and presence of L-NAME did not differ, the mean shift for each segment (mouse) was determined and plotted. ^*^P < 0.05, ^**^P < 0.01, ^***^P < 0.001; 40 vs. 16 mN, *n* = 4.

Levcromakalim, a K_ATP_ channel agonist, clamps the membrane potential of all VSMC to the K^+^ equilibrium potential, and likely obliterates depolarization of the VSMC membrane potential caused by elevated preload (Fransen et al., 2012b). Hence, it was expected that KCC are differently affected at different preloads by levcromakalim-mediated hyperpolarization of the aortic segments. Preloads were set at optimal (16 mN) or physiological preload (40 mN). As expected, levcromakalim shifts the KCC's to higher K^+^ concentrations, irrespective of the presence of L-NAME (Figure 4). With eNOS active, EC_50_ shifted from 32.1 ± 0.8 to 35.4 ± 0.5 mM (*p* < 0.01) at optimal preload, and from 28.3 ± 0.9 to 33.8 ± 0.3 mM (*p* < 0.05) at physiological preload. With eNOS inactive on the other hand, these shifts were respectively from 26.8 ± 0.5 to 30.8 ± 0.4 mM (*p* < 0.001), and from 24.1 ± 1.2 to 28.7 ± 1.0 mM (*p* < 0.05). Although inhibition of NO release by L-NAME caused highly significant shifts of EC_50_ to higher sensitivity, the shifts induced by levcromakalim were comparable whether eNOS was active or inactive. Figure 4C, in which the absolute shifts of EC_50_ by levcromakalim are displayed irrespective of basal NO release, suggests that the shifts caused by repolarization of the membrane potential of VSMC with levcromakalim, are larger at the physiological preload than at the optimal preload and larger at moderate (EC_25_) than at stronger depolarization (EC_50_ and EC_75_).

### α1-adrenergic stimulation with PE

**Figure 5:**
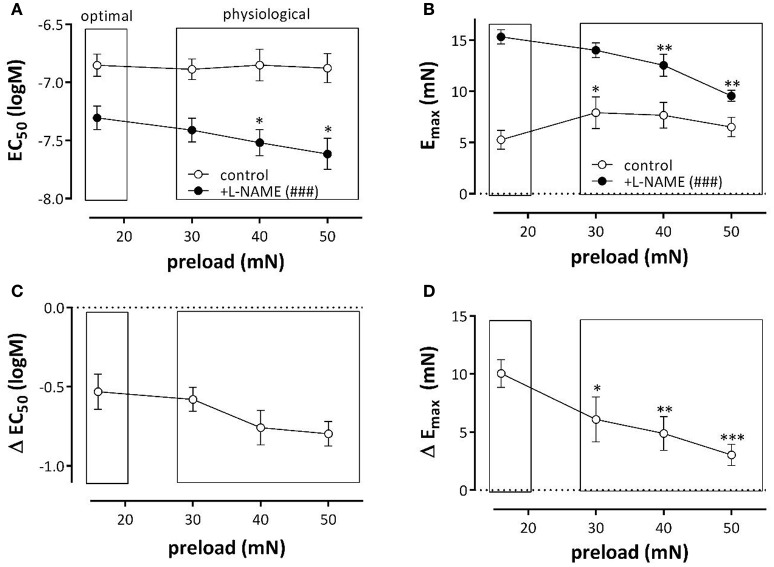
**α1-Adrenoceptor stimulation of aortic segments with phenylephrine at different preloads**. Log(EC_50_) **(A)** and E_max_ **(B)** of PE-induced contractions at different preloads in the absence (control) and presence of L-NAME (+L-NAME, eNOS inhibited). Figures **(C,D)** show the shifts of EC_50_ and of E_max_ by eNOS inhibition with L-NAME. ^*^p < 0.05, ^**^p < 0.01 ^***^p < 0.001: 30, 40, or 50mN vs. 16mN ###*p* < 0.001 control vs. L-NAME.

Similarly as for K^+^, PE concentration-contraction (PECC) curves were constructed at the optimal preload of 16 mN and compared with PECC at 30, 40, and 50 mN. After inhibition of eNOS with L-NAME, the sensitivity of the segments for PE was significantly increased at the physiological preloads when compared with the optimal preload (Figure 5A). However, basal release of NO attenuated this increase in sensitivity and log (EC_50_) of PE was not significantly affected by the different preloads in the absence of L-NAME. As a result the shifts of EC_50_ by eNOS inhibition (Figure 5C) showed a tendency to increase with preload. The role of basal NO release in attenuating PE-induced contractions was even more prominent when considering the maximal effect of PE, which was indeed severely depressed at the optimal preload of 16 mN (Figure 5B). Whereas, 16 mN was the optimal preload for PE-induced contractions in the presence of L-NAME, the optimal preload shifted to 30–50 mN in the absence of L-NAME. Figure 5D demonstrates that ΔE_max_ by L-NAME addition decreased at physiological preloads and, hence, that physiological preloads attenuated the inhibitory effect of NO on PE-elicited contraction observed at the optimal preload. Results suggest that basal NO release protects against preload-dependent changes in the PE-mediated contractions of VSMC.

**Figure 6:**
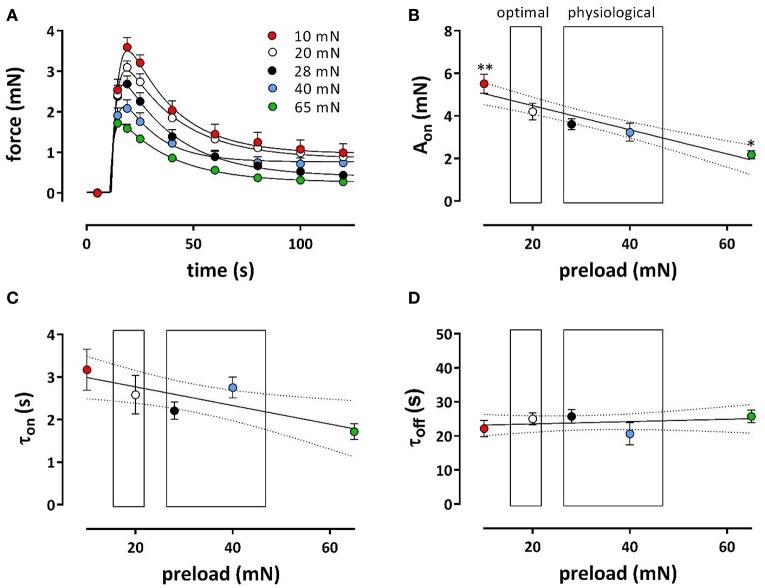
**Phasic contractions of phenylephrine at different preloads**. Contractions induced by 1 μM PE in the presence of L-NAME to inhibit basal release of NO in the absence of extracellular Ca^2+^ at preloads from 10 to 65 mN. Phasic contractions as shown in **(A)** were fitted with a bi-exponential time course revealing amplitudes **(A**_on_, **B)** and time constants (τ_on_, **C**) contraction and time constants of relaxation (τ_off_, **D**). ^*^p < 0.05, ^**^p < 0.01 vs. 20 mN, *n* = 5.

**Figure 7:**
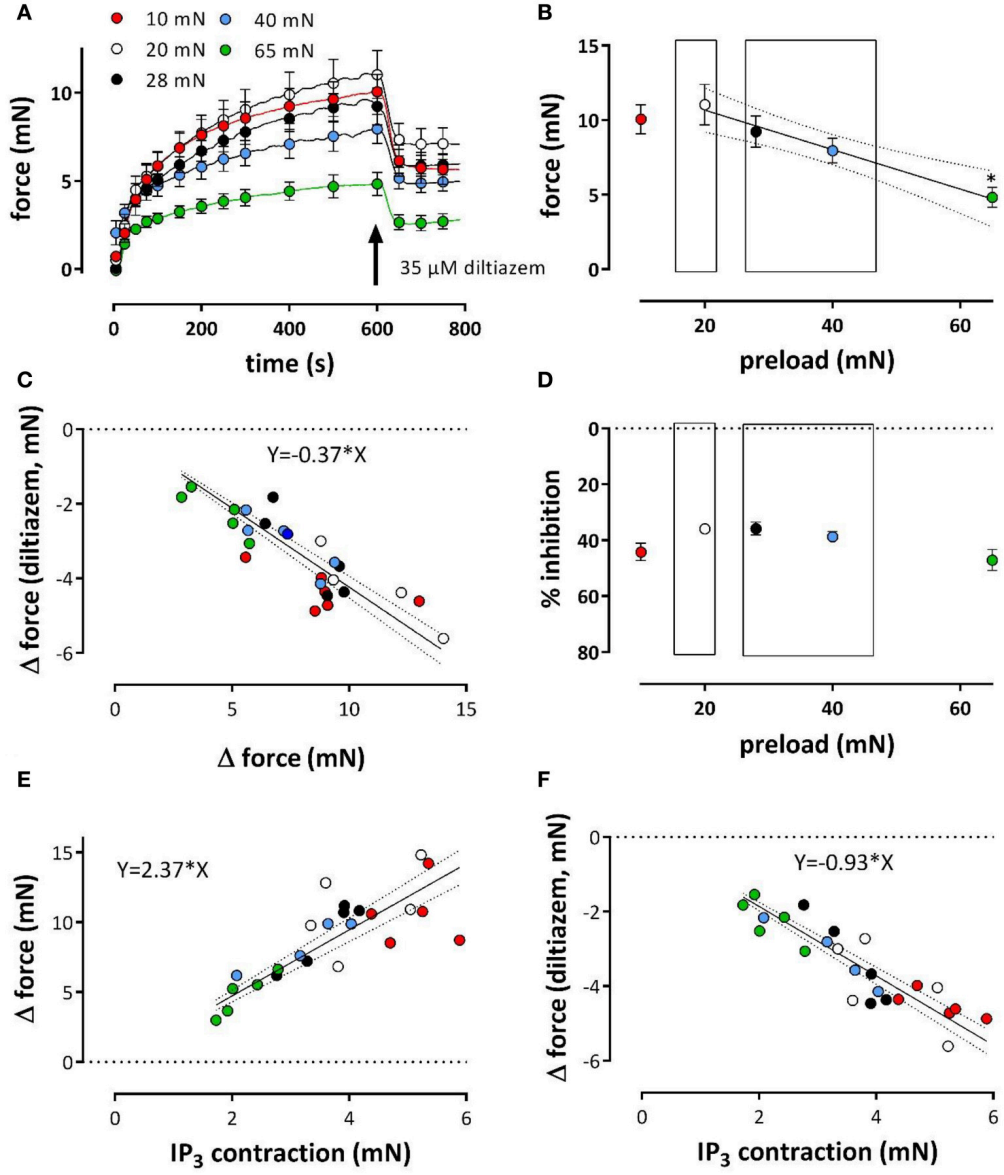
**Tonic contractions of phenylephrine at different preloads**. Contractions were induced at preloads from 10 to 65 mN by re-addition of extracellular Ca^2+^ to segments, which were contracted by 1 μM PE in the absence of external Ca^2+^ (see Figure 6). All contractions were in the presence of L-NAME to inhibit basal release of NO. Tonic contractions after re-addition of external Ca^2+^ as shown in **(A)** were at 600 s inhibited with 35 μM diltiazem. Amplitudes of the contraction at 600 s are shown in **(B)**. In **(C)** the absolute amount of force decrease by diltiazem was plotted against the maximal effect of PE at the different preloads (different colors) and revealed a linear relationship with slope of 0.37. There was a highly significant (*P* < 0.0001) correlation (Pearson correlation *r* = −0.86). Hence, the relative effect of diltiazem **(D)** was load-independent (linear regression line deviates non-significantly from zero). Relationship between PE-induced IP_3_-mediated contraction in the absence of external Ca^2+^ (see Figure 6) and the contraction elicited upon re-introduction of external Ca^2+^ **(E)** or the decrease of this contraction by 35 μM diltiazem (**F**, a measure of the VGCC-mediated component) at different preloads (10 mN: red, 20 mN: white, 28 mN: black, 40 mN: blue and 65 mN: green). There was a highly significant (*P* < 0.0001) correlation (Pearson correlation *r* = 0.79 for **A** and –0.86 for **B**) between both parameters. ^*^p < 0.05 vs. 20 mN, *n* = 5.

Phasic and tonic contractions induced by 1 μM PE in the presence of L-NAME were studied at a broader preload range of 10–65 mN, in the absence of extracellular Ca^2+^ (Figures 6A–D) and after re-addition of external Ca^2+^ (Figures 7A–F). In the absence of external Ca^2+^, the phasic contraction by 1 μM PE, mediated by the IP_3_-dependent release of contractile Ca^2+^ from the SR, was clearly load-dependent (Figure 6A), with a decreasing contractile force at higher load. A double exponential fit of the data revealed that contraction (A_on_, Figure 6B) and relaxation (A_off_, data not shown) amplitude decreased linearly with higher loads. Lower time constants of contraction (τ_on_, Figure 6C) at higher preload suggested that contraction developed faster at higher loads, whereas the time constant of relaxation was load-independent (τ_off_, Figure 6D).

After re-addition of external Ca^2+^, the tonic contraction due to Ca^2+^ influx was maximal at 20 mN, but, as expected from Figure 5B, decreased with higher preloads (Figures 7A,B). To inhibit Ca^2+^ influx via VGCC, 35 μM diltiazem was added after 10 min. Although diltiazem caused smaller relaxations at high preloads, the decrease of force by diltiazem was linearly related with the force induced by PE at the different preloads (Figure 7C). As a consequence, the relative inhibition of the contraction by diltiazem was not dependent on the preload (Figure 7D) and amounted about 35–45% of the total contraction force at all preloads.

By plotting the amplitude of the phasic, IP_3_-mediated contraction (see Figure 6) vs. the contraction in the presence of external Ca^2+^, or vs. the VGCC-mediated contraction (as measured by inhibition of VGCC by diltiazem) at the different preloads, it was observed that a high amplitude of the IP_3_-mediated contraction at low preloads correlated with a high amplitude of the total or VGCC-contraction, and vice versa for the high preloads (Figures 7E,F). These observations might indicate that dependent on the degree of stretch-dependent emptying of the SR Ca^2+^ stores the subsequent contraction by PE via VGCC alone or via VGCC in combination with NSCC is similarly attenuated.

**Figure 8:**
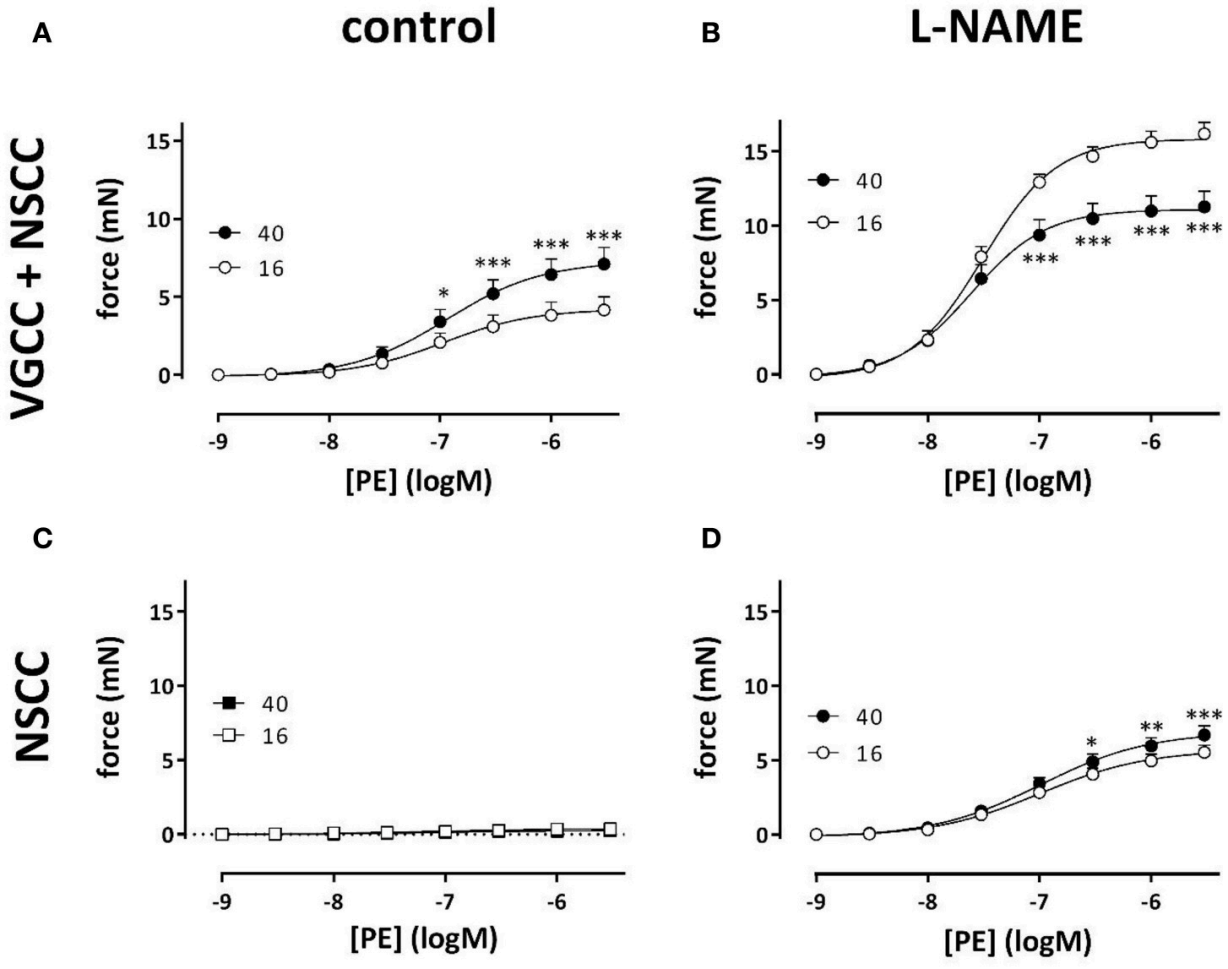
**VGCC and NSCC contribute to PE-mediated contractions at 16 and 40 mN**. PE concentration-response curves for isometric contractions at preloads of 40 mN (black) or 16 mN (white) in the absence (A,C) and presence (B,D) of 300 μM L-NAME were due to Ca^2+^ influx via VGCC and NSCC. Isometric contractions in the presence of 1 μM levcromakalim reveal contractions due to Ca^2+^ influx via NSCC only **(C,D)**. ^*^p < 0.05, ^**^p < 0.01, ^***^p < 0.001, 40 vs. 16 mN preload (*n* = 4).

Results from Figures 3, 4 suggested that VSMC of the aortic segments are depolarized at the higher preload leading to higher sensitivity of the contraction to external K^+^ and increased VGCC Ca^2+^ influx at baseline K^+^. α_1_-Adrenergic stimulation of aortic segments also depolarizes the membrane potential of VSMC, presumably without changing the equilibrium potential for K^+^ ions, and causes contractions which are due to Ca^2+^ influx via VGCC and NSCC (Fransen et al., 2015). With eNOS active, the isometric contractions by PE were significantly larger at physiological (40 mN) than at optimal (16 mN) preload (Figure 8A). After inhibition of basal NO release with L-NAME, however, contractions by PE were substantially increased and, as expected from Figure 5, especially at optimal preload (Figure 8B). Log (EC_50_) of −7.52 ± 0.06 at 16 mN was significantly (*p* < 0.05) less negative than log (EC_50_) of −7.62 ± 0.08 at 40 mN. By adding 1 μM levcromakalim, thereby preventing the load-dependent depolarization of the segments and the PE-induced contractions due to VGCC Ca^2+^ influx, the contraction by PE is due to NSCC Ca^2+^ influx only. In the absence of L-NAME, the contractions were almost completely inhibited by repolarizing the membrane potential of the VSMC with levcromakalim (Figure 8C). As a result, the enhanced isometric contraction by PE at physiological load, as shown in Figure 8A, is completely due to higher Ca^2+^ influx via VGCC, further supporting the hypothesis that segments at physiological preload are depolarized.

**Figure 9:**
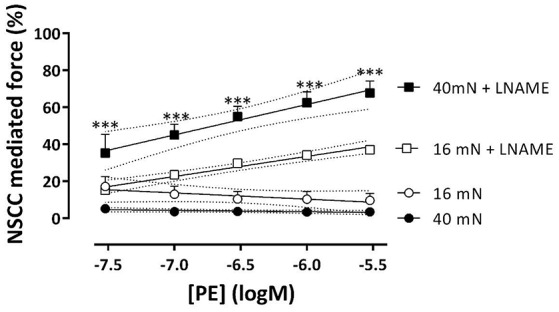
**NSCC-mediated contractions are dependent on NO and preload**. Relative contribution of NSCC-mediated contractions to the total contraction by PE at 16 (white) and 40 mN (black) in the absence (control) and presence of L-NAME to block basal release of NO. ^***^p < 0.001, 40 vs. 16 mN, *n* = 4.

In the presence of L-NAME, repolarizing the segments with levcromakalim did not completely inhibit the contractions by PE as in the absence of L-NAME (Figure 8D). These contractions, which were now due to Ca^2+^ influx via NSCC only, were activated by inhibiting basal NO release with L-NAME, were dependent on the PE concentration and slightly, but significantly higher at physiological than at optimal preload (Figure 8D). The log (EC_50_) of the PECC were load-independent (−6.99 ± 0.04 logM both at 16 and 40 mN), and were significantly less negative than in the absence of levcromakalim at 16 (*p* < 0.05) and 40 (*p* < 0.01) mN (data not shown). These results suggested that aortic segments were far more sensitive to PE when contraction was elicited via VGCC than via NSCC. As a result, the relative contribution of NSCC to the total contraction increased with the PE concentration as shown in Figure 9. Thereby, the relative contribution of the NSCC-mediated contractions to the total contraction was significantly larger at 40 than at 16 mN at each PE concentration.

### Relaxation by endogenous and exogenous NO of isometric contractions induced by phenylephrine at different preloads

**Figure 10:**
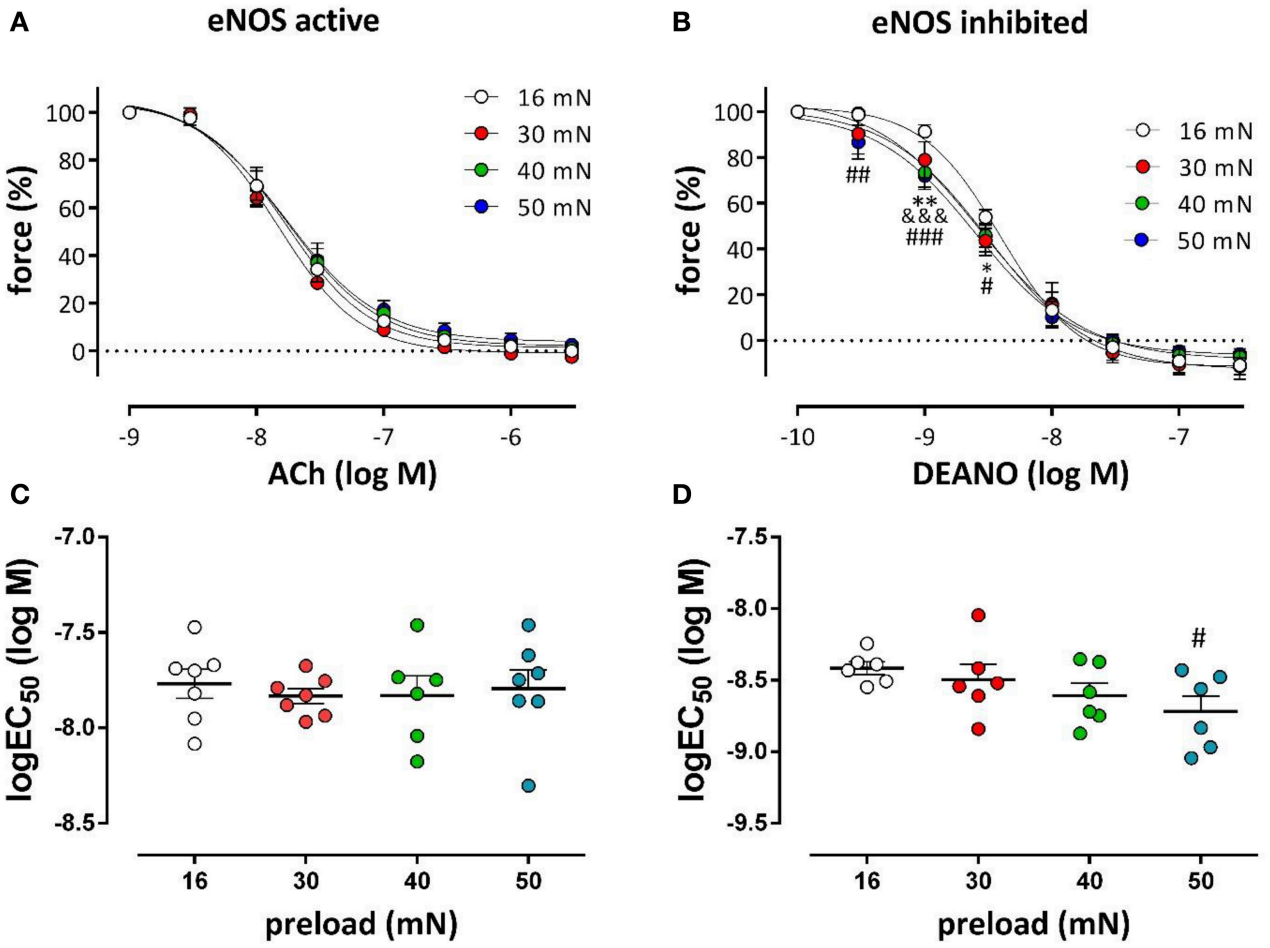
**Relaxation of PE-precontracted aortic segments by endogenous and exogenous NO at different preloads**. DRCs for ACh- **(A)** and DEANO- **(B)** induced relaxations at the different preloads. Pre-contractions were elicited with the EC50 concentration of PE in the absence **(A)** and presence **(B)** of 300 μM L-NAME. The log(EC50)-values of ACh **(C)** and DEANO **(D)** were calculated at the different preloads (*n* = 6); ^*^P < 0.05, ^**^P < 0.01, 16 vs. 30 mN; ^&&&^*P* < 0.001, 16 vs. 40 mN; ^#^*P* < 0.05, ^##^*P* < 0.01, ^###^*P* < 0.001, 16 vs. 50 mN.

Aortic segments, which were mounted at the different preloads, were pre-contracted with the half maximal effective concentration of PE in the absence and presence of L-NAME and relaxed by endogenous (ACh) or exogenous (DEANO) NO respectively (Figure 10). For endogenous NO, there were no differences in the relaxation curves for the different preloads, indicating normal ACh-mediated NO release by the EC. Exogenous NO, however, caused larger relaxations especially at the lower DEANO concentrations and the NO-sensitivity of the VSMC at the higher preloads was increased. Log (EC_50_)-values for ACh-relaxations were preload-independent (Figure 10C), whereas these for DEANO were preload-dependent (Figure 10D).

## Discussion

The present study showed that with higher than optimal preloads mouse aortic segments were depolarized, the contraction due to SR contractile Ca^2+^ release decreased, contractions via voltage-dependent Ca^2+^ channels were decreased but more sensitive to depolarization and contractions via NSCC Ca^2+^ influx were increased and very sensitive to the basal release of NO.

### Interaction between passive and active components

The inner diameter of mouse and rat aorta is nearly linearly related to intraluminal pressures between 0 and 120 mmHg, but deviates from linearity at higher pressures (Rapacon-Baker et al., 2001; Wu et al., 2014). In the present study, passive aortic segment diameters were linearly related with preloads up to 40 mN, but were smaller than expected from this relationship at 50 mN preload (Figure 1). This has important consequences for active force development. As expected from the “definition” of optimal preload, the maximal contractions by depolarization with K^+^ in the absence and presence of L-NAME decreased with elevated stretch of aortic segments above optimal preloads. α_1_-Adrenoceptor stimulation with PE after eNOS inhibition with L-NAME caused a similar stretch-dependency of the maximal contractions. These observations indicate that the attenuated contraction of maximally depolarized or α_1_-adrenoceptor stimulated VSMC with increased stretch is due to a stretch-dependent increase in the resistance of passive vessel components. In mouse aortic segments vessel compliance decreases with VSMC contraction and with higher pressures, compatible with the anisotropic nature of the aortic wall and the non-linear stress-strain relations (Vayssettes-Courchay et al., 2011; Leloup et al., 2016; Murtada et al., 2016). With maximal effects of about 15 mN by 50 mM K^+^ or 1 μM PE, this means that the deviation from linearity will occur at lower preloads when segments are actively stimulated with K^+^ or PE and that the E_max_ has to decrease with preload in these conditions.

### Stretch depolarizes mouse aortic segments

In hypertensive rats, increments of pressure above 100 mmHg cause a pressure- and stretch-activated constrictor response that relies on increased calcium influx through voltage-dependent Ca^2+^ channels. This phenomenon leads to decreased diameters when compared with normotensive rats (Rapacon-Baker et al., 2001). In mesenteric arteries of hypertensive rats, it has been described that the density of VGCC Ca^2+^ current, and large conductance Ca^2+^-dependent K^+^ current as well, are elevated, since the sensitivity to nifedipine and the Ca^2+^ dependent K^+^ channel agonist NS11021 is increased (Shi et al., 2015). Comparable results were obtained in Angiotensin II-treated hypertensive mice (Fransen et al., 2016). Moreover, hypertension depolarized VSMC from −50 to −37 mV (Pesic et al., 2004). These chronic effects of hypertension, which are paralleled by structural changes in the blood vessel wall, cannot be compared, however, with the acute effects of increased preload described in the present study (Fridez et al., 2002).

As observed in hypertension (Pesic et al., 2004), the present study indicated that an acute increase of preload or stretch also depolarizes aortic VSMC at least in the absence of a functional endothelium. This was supported by the following observations. (1) KCC curves revealed that the EC_50_ at the physiological preloads was shifted to lower K^+^ concentrations than at the optimal preload (Figure 2). (2) Repolarization of the membrane potential of VSMC with levcromakalim caused shifts of the EC_50_ for external K^+^, which were L-NAME independent, which were larger at the physiological than at the optimal preloads and which were larger at moderate (EC_25_) than at stronger depolarization (EC_50_ and EC_75_) (Figure 4). The latter can be explained by the larger deviation of the resting potential of the aortic VSMC from the K^+^ equilibrium potential at normal or moderate than at high (50 mM) K^+^ concentrations (Fransen et al., 2012a,b). Removal of extracellular Ca^2+^ caused a larger decrease of basal tonus at the higher than at lower preloads (Figure 3). In conditions of depolarized VSMC baseline Ca^2+^ influx via VGCC is expected to be larger (Leloup et al., 2015a). Also in WKY rat aorta segments (1 mm) an increase of stretch tension from 8 to 50 mN caused increased spontaneous tone, especially after inhibiting basal release of NO (Sekiguchi et al., 2001). (4) In the presence of L-NAME, the EC_50_ for PE shifted to lower PE concentrations at higher preloads (Figure 5).

### Repolarizing action of NO

NO has been described to activate voltage-gated K^+^ channels, to hyperpolarize VSMC and to decrease their intracellular Ca^2+^ concentration (Yuan et al., 1996; Quignard et al., 2000; Van Hove et al., 2009; Edwards et al., 2010). Aortic segments release considerable amounts of NO in basal conditions (van Langen et al., 2012; Leloup et al., 2015b). Nevertheless, this high basal NO release had only minor effects on maximal contractions induced by depolarization at the different preloads. Indeed, similarly as for levcromakalim, the hyperpolarizing effect of NO is largely dependent on the external K^+^ concentration and is completely suppressed for depolarization by high 50 mM K^+^. On the other hand, the hyperpolarizing effect of basal NO release causes significant shifts of the EC_50_ of external K^+^ to higher K^+^ concentrations, which were nearly preload-independent (Figure 2). As contractions by depolarization are mainly due to Ca^2+^ influx via VGCC (Fransen et al., 2012a), these observations suggest that basal release of NO is preload-independent and affected contractions via VGCC at the different preloads mainly via its repolarizing effect on the VSMC and not by direct inhibition of the VGCC. We have previously shown that the relaxing effect of ACh is dependent on the extracellular K^+^ concentration and increased from about 10–60% for a K^+^ decrease of 50–20 mM (Van Hove et al., 2009), which indicates that the repolarizing effect of NO is indeed larger at moderate than at larger depolarization.

E_max_ and log (EC_50_) by α_1_ stimulation with PE were nearly load-independent between 16 and 50 mN, in the presence of basal NO release (Figure 2). Hence, a functional endothelium protected the VSMC of aortic segments against their inherent preload-dependency or, vice versa, endothelial dysfunction implies load-dependency to the aortic segments, which become more sensitive to PE, but display lower contractility at higher preloads. This might again be explained by the hyperpolarizing action of NO.

### Stretch and VSMC

In the acutely stretched mouse thoracic aorta, two types of stretch-elicited VSMC Ca^2+^ responses were observed, one was a phasic calcium discharge generated by the SR, the second was a tonic response produced by the activation of stretch-sensitive cationic channels allowing external Ca^2+^ entry (Fanchaouy et al., 2007). The phasic, IP3-mediated contraction at 1 μM PE in the absence of external Ca^2+^, as shown in Figure 6 and mediated by Ca^2+^-release from the SR (Fransen et al., 2015), decreased with higher load confirming a Ca^2+^ discharge from the SR by stretch. Moreover, the time constant of contraction (τ_on_, Figure 6B) was smaller at higher loads, suggesting that IP_3_-sensitive SR Ca^2+^ stores release their Ca^2+^ content faster at higher stretch. Results indicate that the contractile Ca^2+^ stores of the SR of VSMC in the mouse aorta are extremely stretch-dependent. Previously, we have described that the IP_3_-mediated PE-induced contraction was dependent on baseline Ca^2+^ influx via VGCC (Leloup et al., 2015a). If stretch depolarizes the VSMC, one should expect larger IP_3_-mediated contractions at high preload, which was, however not observed. Results suggest that there is a direct effect of preload on the IP_3_-sensitive SR Ca^2+^ store with higher preloads emptying the stores. There was a highly significant, linear relationship between the VGCC-mediated component of the PE-induced contraction and the phasic IP3-mediated contraction, which might indicate that the PE-induced contraction is dependent on the amount of Ca^2+^ that can be released from the IP_3_-sensitive SR Ca^2+^ store.

Because the inhibition of the PE-elicited contraction by the VGCC blocker diltiazem was linearly related to the total PE-contraction (Figure 7D), it was concluded that the relative contribution of the VGCC or NSCC component of these contractions was preload-independent. However, in the presence of levcromakalim, the VGCC and NSCC component of the PE-induced contraction were stretch-dependent with the VGCC component decreasing and the NSCC component increasing with higher preload (Figure 8). Because levcromakalim repolarizes the VSMC to negative membrane potentials and thus prevents the depolarizing effect of increased preloads and concomitant activation of VGCC, stretch of the aortic segment by increasing preload or PE concentration, favors the NSCC-mediated contraction at the expense of VGCC-mediated contractions.

In the present study, basal release of NO mainly restricts the contraction by PE by inhibiting the Ca^2+^ influx via NSCC (Cohen et al., 1999). In the presence of levcromakalim, eNOS inhibition with L-NAME increased PE-elicited force substantially and more at physiological than at optimal preload.

There were no differences in the ACh-induced relaxation for the different preloads, indicating normal ACh-mediated NO release by the EC. Exogenous NO, however, caused larger relaxations especially at the lower DEANO concentrations and NO-sensitivity of the VSMC was increased at the higher preloads. It should be kept in mind, however, that DEANO was applied to segments treated with L-NAME, which causes a large increase in contraction via NSCC, which are very sensitive to NO. If at high preload, NSCC-mediated contractions are stimulated, it is expected that these are very NO-dependent, leading to load-dependency of log (EC_50_)-values for DEANO. Log (EC_50_)-values for ACh were independent (Figure 10C), probably because NSCC are inhibited in the presence of basal NO release and PE-induced contractions via VGCC are preload-independent in the absence of L-NAME.

In conclusion, the present study demonstrates that at preloads in the physiological range, VSMC of mouse aortic segments are depolarized, have a lower SR Ca^2+^ content, increased contraction via NSCC, and unchanged contractions via VGCC. The fact that segments at higher preloads are depolarized has important physiological consequences in that these segments can both constrict as well as dilate in basal conditions, dependent on the experimental conditions (membrane potential). Clinically, this increased VSMC membrane potential translates to a higher basal tone of the thoracic aorta. This will further result in increased aortic stiffness, as indicated by both experimental (Fridez et al., 2001; Gao et al., 2014; Leloup et al., 2016) and clinical studies (Wilkinson et al., 2002; Bia et al., 2008). The results of this study further indicate that basal NO release mainly affects NSCC-mediated contractions, but seemed to be preload-independent. Moreover, at high preloads, endothelial release of NO protects against the preload-dependency of α-adrenergic VSMC contractions. This has major repercussions for patients with endothelial dysfunction, a common symptom of cardiovascular disease, for whom this compensation mechanism is deficient, leading to an increased sensitivity to the circulating catecholamines, again increasing the basal VSMC tone. Lastly, this study holds a clear warning about the extrapolation of vasoreactivity data obtained at an optimal preload. This study clearly demonstrates that physiological changes in mouse aortic segments, measured at subphysiological preloads, do not necessarily represent the clinical *in vivo* situation, especially for pathological conditions, such as hypertension.

## Author contributions

SD, AL, CV, and PF: Design of research, performed experiments, analyzed data, interpreted results of experiments, drafted manuscript, and prepared figures; SD, AL, CV, GD, and PF: Approved final version of manuscript and accountability for accuracy and integrity of the work.

### Conflict of interest statement

The authors declare that the research was conducted in the absence of any commercial or financial relationships that could be construed as a potential conflict of interest.
